# Risk Factors Associated With Hearing Impairment in Infants and Children: A Systematic Review

**DOI:** 10.7759/cureus.40464

**Published:** 2023-06-15

**Authors:** Waleed Alhazmi

**Affiliations:** 1 Department of Otolaryngology-Head and Neck Surgery, Qassim University, Buraydah, SAU

**Keywords:** universal newborn hearing screening, neonatal intensive care unit, jcih risk factors, hearing loss, auditory brainstem response

## Abstract

The purpose of the present systematic review was to synthesize evidence on associated risk factors of hearing loss (HL) in children.

Evidence-based research articles on HL published between January 2013 and December 2022 using PubMed, Cochrane, and Scopus databases were searched. The study included children between zero and three years of age who have permanent bilateral/unilateral HL (BHL/UHL) by employing case-control studies, randomized controlled trials, nonrandomized studies, prospective or retrospective cohort studies, and studies with or without comparison groups. The Newcastle-Ottawa Scale (NOS) and the Joanna Briggs Institute (JBI) critical appraisal checklist for longitudinal and cross-sectional studies were used to rate the quality of the chosen studies. The studies that would be considered were reviewed by two independent authors, and a third author was contacted if there was a dispute.

A preliminary literature search uncovered 505 articles from the electronic search and 41 studies by hand searching. Duplicate records were eliminated, leaving 432 records. The abstract and title were read, and 340 studies were eliminated. There were 92 articles in total that qualified for full-text screening. Among these, 75 articles were disregarded since they lacked information or failed to assess the risk factors for HL. The qualitative synthesis, therefore, included 17 articles. Most often, cross-sectional study designs were used in the studies that were reviewed, which were then followed by longitudinal studies. Three of the studies that were reviewed used a prospective cohort design. The quality of all the included studies was rated to be good.

The current review revealed that the primary statistically significant risk factors for HL included ventilator support; craniofacial anomalies; low birth weight (LBW); forceps delivery; loop diuretics; meningitis; asphyxia; intensive care; consanguinity; sepsis; Apgar scores between 0 and 4 at one minute; toxoplasmosis, other agents, rubella, cytomegalovirus, and herpes (TORCH) infections; and hyperbilirubinemia.

## Introduction and background

Permanent childhood hearing disorder is characterized as a proven permanent bilateral hearing impairment of more than 40 dBHL averaged over the frequency range of 0.5, 1, 2, and 4 kHz in the effective hearing ear. It can be mainly ascribed to genetic and environmental factors, implying that it can be congenital or of acquired origin. In accordance with the 2018 World Health Organization (WHO) projections, children contribute to 7% of all individuals globally with hearing loss (HL) [1]. It constitutes one of the most common sensory dysfunctions in newborn babies, with a reported incidence ranging from 0.5 to five per 1000 cases globally. Permanent congenital hearing loss is 10-20 times more common in newborns hospitalized in the neonatal intensive care unit (NICU) compared to the general population [2]. The high prevalence mandates careful observation as it is well known that the initial 36 months after birth are a pivotal period for cognitive and language development [3].

Children with genetically inherited and early-onset hearing loss are detected clinically and endorsed to learn language during the first few months of life through the implementation of universal newborn hearing screening (UNHS) initiatives as a component of a comprehensive early hearing detection and intervention (EHDI) program [4]. The majority of developed nations have implemented UNHS programs, which are described as universal screening by the age of six months with otoacoustic emission (OAE) tests, auditory brainstem responses (ABR), or both, accompanied by diagnostic referral when necessary [5]. One of the following risk factors outlined affects approximately 50% of children with permanent congenital HL: family history of HL, NICU care, perinatal infection, low birth weight (LBW), asphyxia, craniofacial malformations, hyperbilirubinemia, and chromosomal aberrations. Apgar scores between 0 and 4 at one minute are considered the second most significant risk factor, followed by toxoplasmosis, other agents, rubella, cytomegalovirus, and herpes (TORCH) infections [2].

According to the WHO, chronic middle ear infections are thought to be the preventable cause of 60% of HL in children [6]. Congenital or perinatal HL should indeed be identified within three months of birth for successful therapy, with a definitive diagnosis, and EHDI should be initiated before the child turns six months old [7]. There is a dearth of a comprehensive or organized systematic review of the risk factors for HL in children. Moreover, identifying the risk factors that are most likely to result in HL in infants can be beneficial in the planning and implementation of preventive strategies, focused on modifiable risk factors [2]. The purpose of the present systematic review was to synthesize evidence on associated risk factors of HL in children.

## Review

Materials and methods

The Preferred Reporting Items for Systematic Reviews and Meta-Analyses (PRISMA) statement for the report was adhered to in the study protocol. The structured question designed for the review was the following: "What are the environmental and demographic factors (E) significantly related to congenital-, early-, or delayed-onset HL (O) in children (P)?"

Information Sources and Search Strategy

Evidence-based research articles on HL published between January 2013 and December 2022 using PubMed, Cochrane, and Scopus databases were searched. Medical Subject Heading (MeSH) terms were used. After an initial screening of the literature, the terms ("Hearing loss" OR "Hearing impairment" OR "Hearing Deficit" OR "Auditory Deficit") AND ("Risk Factors" OR "Demographic Factors" OR "Environmental Factors" OR "Maternal Risk Factor") AND ("Infant Hearing" OR "Permanent Childhood Hearing Loss" OR "Universal Newborn Hearing Screening" OR "Sensorineural" OR "Conductive Hearing Disorder" OR "Congenital Hearing Loss" OR "Unilateral Hearing Loss" OR "Bilateral Hearing Loss" OR "Joint Committee on Infant Hearing") AND ("Apgar Score" OR "Otoacoustic Emissions" OR "Auditory Brainstem-Response" OR "Tympanometry" OR "Audiometry" OR "Ototoxic Drugs") were used as keywords. Additional manual research was also carried out. Furthermore, the electronic search of the databases was also conducted through the reference lists of the included articles. The following evaluation did not include duplicate articles, non-English-language articles, or articles that did not specifically address the risk factors associated with HL.

The population, intervention, comparison, and outcome (PICO) for our research question is as follows.

Population: The study will include children of age 0-3 years who have permanent bilateral/unilateral HL (BHL/UHL).

Exposure: Conditions such as congenital cytomegalovirus, toxoplasmosis, LBW, admission to a NICU, ototoxic drugs, hyperbilirubinemia, meningitis, sepsis, craniofacial malformations, and a family history of HL are the primary statistically significant risk factors for HL. Risk elements related to transient conductive hearing loss (CHL) will be disregarded.

Comparison group: The comparison group either lacked any risk factors or compared one risk factor to other factors. The presence of a control group was flexible given the nature of studies in order to gather thorough data.

Outcome measures: The study included those with permanent BHL or UHL in childhood, which could be of the conductive, sensorineural, or mixed type. Studies that addressed pathologies of auditory processing, however, were not included.

Study design: The following study types will be considered to the extent that they address the research question: case-control studies, randomized controlled trials, nonrandomized studies, prospective or retrospective cohort studies, and studies with or without comparison groups. Case reports or case studies, research on hearing impairment in elderly individuals, and gray literature such as unpublished reports, theses, or qualitative research will be refrained from being included in this review.

Selection of Studies, Data Collection, and Data Extraction Process

**Figure 1:**
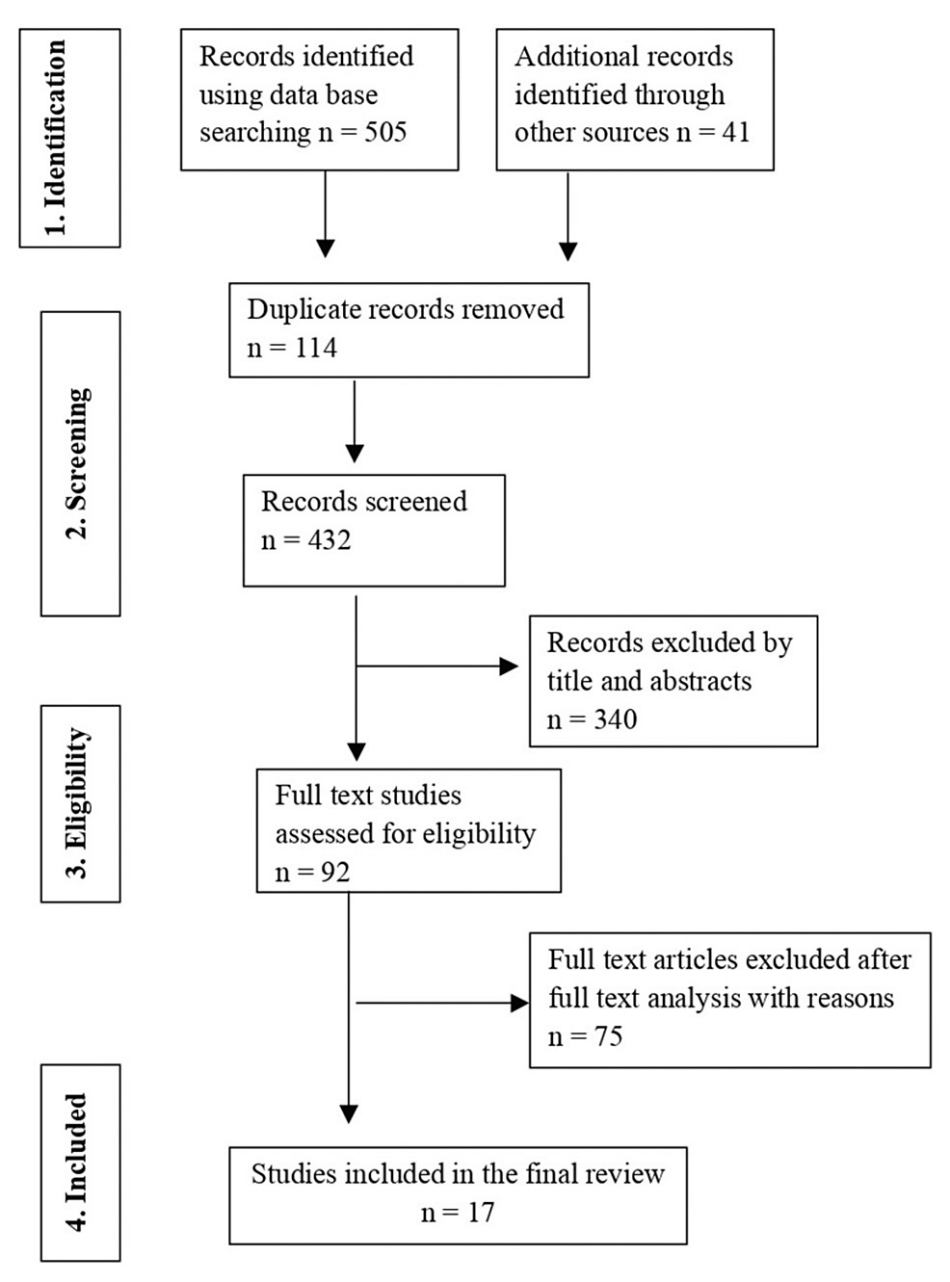
Preferred Reporting Items for Systematic Reviews and Meta-Analyses (PRISMA) flow chart of the included studies Adapted from Preferred Reporting Items for Systematic Reviews and Meta-Analyses 2009 flow diagram

Studies that met the inclusion criteria were selected for full-text review after being assessed depending on their title or abstract. The studies that would be considered were reviewed by two independent authors, and a third author was contacted if there was a dispute. The search and selection strategy is shown in Figure 1.

The name and country of the author, publication year, study design, sample size, sample population, study tool used to assess hearing, outcome measures, statistical analysis applied, and implications of the reviewed studies were retrieved in a predetermined table. The corresponding authors of each article were contacted if there was a disparity in the information obtained.

Risk of Bias (RoB) of Individual Studies

The Newcastle-Ottawa Scale (NOS) and the Joanna Briggs Institute (JBI) critical appraisal checklist for longitudinal and cross-sectional studies, respectively, were used by two independent reviewers to rate the quality of the chosen studies. Eight items make up the NOS, which assesses four factors such as sample selection and representativeness, comparability, and outcome evaluation. Apart from the purview of comparability, which receives a maximum of two stars, each item is given a maximum of one star. A study with more stars is of greater quality [8].

Eight criteria are evaluated by the JBI critical appraisal guideline: sample selection criteria, subject characterization, measurement of exposure, measurement of subject condition, identifying confounding factor, control of confounding factor, outcome evaluation, and statistical analysis. There are four categories for each item: yes, no, unclear, and not applicable [9]. Any conflicts that arose during the selection of the data and the evaluation of its quality were resolved through discussion involving a third reviewer.

Results

Study Selection

The PRISMA flow diagram was used to guide the article review and data extraction processes (Figure 1). A preliminary literature search uncovered 505 articles from the electronic search and 41 studies by hand searching. Duplicate records were eliminated, leaving 432 records. The abstract and title were read, and 340 studies were eliminated. There were 92 articles in total that qualified for full-text screening. Among these, 75 articles were disregarded since they lacked information or failed to assess the risk factors for HL. The qualitative synthesis therefore included 17 articles [10-26].

Table 1 provides an overview of the studies, methods, sample size, sample population, assessment tools, statistical analysis, and results. Most often, cross-sectional study designs were used in the studies that were reviewed [10,11,13,15,16-19,21,23-26], which were then followed by longitudinal studies [12,14,18,20,22]. Three of the studies that were reviewed used a prospective cohort design [10-12].

**Table 1:** Summary characteristics of the reviewed studies HL, hearing loss; OAE, otoacoustic emissions; DPOAE, distortion product otoacoustic emissions; AABR, automated auditory brainstem responses; TEOAE, transient-evoked otoacoustic emissions; ABR, auditory brainstem responses; DEOAE, distortion-evoked otoacoustic emissions; BERA, brainstem-evoked response audiometry; BOA, behavioral observation audiometry; VRA, visual reinforcement audiometry; AHEMD-IS, affordances in the home environment for the motor development-infant scale; ASSR, auditory steady-state responses; UNHS, universal neonatal hearing screening; NICU, neonatal intensive care unit; SNHL, sensorineural hearing loss; CHL, conducting hearing loss; MHL, mixed hearing loss; BHL, bilateral hearing loss; UHL, unilateral hearing loss; RIHL, risk indicators for hearing loss; TORCH, toxoplasmosis, other agents, rubella, cytomegalovirus, and herpes; NNE, neonatal neurological examination; PDA, patent ductus arteriosus; ROP, retinopathy of prematurity; IVH, intraventricular hemorrhage

Author and year	Country	Study design and follow-up duration	Sample population and sample size	Study tool used for measuring hearing	Statistical analysis	Study implications
Hajare and Mudhol, 2022 [10]	India	Prospective cross-sectional study	There were 402 in the NICU and 396 well baby nursery babies	DPOAE and AABR	Frequency distribution, chi-square test, and receiver operating characteristic curve	Family history of deafness, consanguineous marriage (p=0.003), anemia and hypertension in antenatal care (ANC), TORCH in mother (p = 0.022), low Apgar score, and hyperbilirubinemia (p = 0.001) in newborns were a major risk factor
Omar et al., 2022 [11]	Egypt	Prospective cross-sectional study between March 2020 and January 2021	Two out of 200 cases (1%) had HL	TEOAE	Logistic regression analysis	There was a statically significant effect of the prematurity alone on the HL (p < 0.037). The combination of preterm and low birth weight was also statistically significant (p < 0.006)
Salvago et al., 2022 [12]	Italy	Prospective cohort study with a mean follow-up of 20.11 ± 1.69 months	Children with a mean age of four weeks admitted to NICU comprising 338 with normal hearing and 40 with SNHL	ABR, TEOAE, tympanometry, BOA, or VRA	Simple logistic regression analysis	The frequency of extremely preterm and extremely low birth weight (p < 0.05) and prenatal (4.49%) and peri-natal infections (9.52%) (p < 0.0001) were more in the SNHL group. Simple logistic regression analysis showed statistically insignificant association between prematurity (CI = 0.89-1.33; p = 0.33) and very low birth weight (CI = 0.98-1.00; p = 0.19). Children with worse hearing thresholds of initial ABR were less likely to exhibit auditory maturation (CI = 0.95-0.99; p = 0.02)
Zaqqout and Hamad, 2022 [13]	Gaza governorates	Case-control study conducted between February 2017 and July 2018	Children of less than three years of age (n = 338 with 1:1 ratio of cases and controls)	Face-to-face household interviews	Logistic regression analysis	Cases are children with varying degrees of SNHL. The following risk factors were more prevalent in cases: family history and consanguinity (p = 0.001), lack of antenatal care (OR = 0.341; 95% CI = 0.181-0.640; p = 0.002), prematurity (p = 0.006), low birth weight (p < 0.0001), NICU (p = 0.002), recurrent otitis media (p < 0.0001), and exposed to sporadic loud noises (p = 0.01)
Jeong et al., 2021 [14]	Korea	Retrospective case-control study of children born between 2007 and 2013 and that were followed up until 2015	Children with hearing disability (n = 847) and control (n = 2508)	Data retrieved from the National Health Insurance Service of Korea	Multivariate regression analysis	Accompanying brain lesions (OR = 24.87; 95% CI = 9.28-6.66), ototoxic drugs such as aminoglycosides or loop diuretics (OR = 2.58; 95% CI = 1.64-4.06), NICU admission for more than five days (OR = 2.98; 95% CI = 1.62-5.51), and a maternal disability at delivery (OR = 15.91; 95% CI = 6.38-39.70)
Choi et al., 2020 [15]	Korea	Retrospective cross-sectional study	NICU infants (n = 2404) who had been performed UNHS from 2004 to 2017. The study group consists of 43 HL cases and 172 in the control group	ABR	Pearson chi-square test and Student's t-test	History of sepsis, peak total bilirubin, duration of vancomycin use, days of phototherapy, and exposure to loop-inhibiting diuretics were found to be significant risk factors
Gohari et al., 2020 [16]	Iran	Cross-sectional study	Out of 203 NICU infants within 24 months of birth, 159 had HL, and 44 were normal	TEOAE, AABR, and ABR	Chi-square test	Of the sample, 5.66% was identified with different types of HL, 2.51% had SNHL, 0.62% had auditory neuropathy, and 2.51% had CHL. Birth weight of less than 1500 g, hyperbilirubinemia, antibiotic therapy, family history of hearing loss, asphyxia, and Apgar score of less than 5 were significant risk factors
Hardani et al., 2020 [17]	Iran	Cross-sectional study of infants born between August 2019 and April 2020	53 NICU infants with mean age of 6.65 ± 6.96 days	In infants with abnormal AABR and TEOAE results, ABR and ASSR tests were performed.	Frequency distribution for descriptive statistics and chi-square test	5.09% were diagnosed with different types of hearing loss. Ototoxic drugs, hyperbilirubinemia requiring exchange transfusion, asphyxia, low weight birth, Apgar score of <5, and a kinship marriage of parents were significant risk factors
Niu et al., 2020 [18]	Stockholm, Sweden	Retrospective cohort study of children with HL born between January 2009 and December 2013	Children between the age of six months and 5.5 years were enrolled. The mean age at the time of referral was 13.2 ± 14.3 months comprising 221 with bilateral hearing loss and 75 with unilateral hearing loss	UNHS using multiple recordings of TEOAE followed by clinical ABR	Percentage distribution of risk factors using Excel	Etiology was identifiable in 93 children with BHL, wherein syndromic HL accounted for 37.2%, chromosomal aberrations for 21.3%, and environmental causes for 19.1%. In UHL, etiology was ascertained in 35 cases, wherein ear malformation was more frequent (74.3%), followed by environmental causes (14.3%)
Araujo et al., 2019 [19]	Brazil	Cross-sectional study	Infants between eight and 10 months with 77 RIHL (study group) and 77 without RIHL (control group)	OAE and AHEMD-IS	Mann-Whitney test and the chi-square test	A statistically significant difference in the total score of the groups (p = 0.013) was observed. The homes of infants with risk indicators for hearing loss have statistically less affordances when compared to the homes of infants without the indicators
Labaeka et al., 2018 [20]	Nigeria	Longitudinal cohort study between November 2014 and February 2015	201 newborns in the neonatal unit with risk factors for hearing impairment and 134 had HL	AABR	Multivariate logistic regression analysis	The majority of hearing loss at follow-up was bilateral (94.7%) and severe (52.6%). The risk factors associated with persistent hearing loss were acute bilirubin encephalopathy (RR = 11.2; 95% CI = -1.4-90.6), IVH (RR = 8.8; 95% CI = 1.1-71.8), meningitis (RR = 4.8; 95% CI = 1.01-29), recurrent apnea (RR = 2.7; 95% CI = 1.01-7.3), and severe perinatal asphyxia NNE III (RR = 7; 95% CI = 2.4-20.2)
Wroblewska-Seniuk et al., 2018 [21]	Poland	Retrospective study of children born between January 2010 and December 2013 with hearing deficit	Children with SNHL (n = 38), CHL (n = 56), and MHL (n = 15).	UNHS using OAE examined by means of ABR method	Pearson chi-square and Kruskal-Wallis tests	Hyperbilirubinemia predisposes to SNHL (p < 0.05). Isolated craniofacial malformations were found to be associated with CHL (p < 0.05).
Poonual et al., 2016 [22]	Northern Thailand	Prospective cohort study between November 2010 and May 2012	3120 infants of three-month-old age were screened, and 135 had HL	UNHS using automated OAE	Multivariate regression risk analysis	The following were the reported risk factors for the bilateral hearing loss: birth weight of 1500-2500 g (RR = 1.6; 95% CI = 1.1-2.6; p = 0.02), Apgar score of 6 at five minutes (RR = 2.2; 95% CI = 1.1-4.4; p = 0.02), craniofacial anomalies (RR = 2.5; 95% CI = 1.6-4.2; p < 0.001), sepsis (RR = 1.8; 95% CI = 1.0-3.2; p = 0.04), and ototoxic exposure (RR = 4.1; 95% CI = 1.9-8.6; p < 0.001)
Abu-Shaheen et al., 2014 [23]	Jordan	Cross-sectional study conducted between July 2007 and 2008	63041 infants were screened, and 1103 were referred. With HL, 966; without HL, 97; dropouts, 40	DPOAE	Multivariate logistic regression	The presence of at least one Joint Committee on Infant Hearing (JCIH) risk factor, admission to NICU for more than five days (p = 0.027), age, birth weight (p < 0.01), postnatal hypoxia (p = 0.004), and forceps delivery (p = 0.034) were independently associated with hearing loss. Four of these six factors are not on the JCIH factors: age, birth weight, postnatal hypoxia, and forceps delivery. Infants with at least one of the 10 JCIH risk factors for hearing loss had a 1.7-fold increased risk for hearing loss compared with infants without any risk factors (95% CI = 1.43-4.85)
Eras et al., 2014 [24]	Turkey	Retrospective cohort between September 2009 and December 2011	Preterm infants (N = 1360) born with a gestational age of less than 32 weeks and/or birth weight of less than 1500 g hospitalized in the NICU. 33 infants were referred, and 19 had HL	Clinical OAE, multifrequency and conventional tympanometry, and diagnostic ABR testing	Multinominal logistic regression analysis	The significant factors related to HL include proven sepsis (OR = 5.5; 95% CI = 1.01-16.3; p = 0.019), mechanical ventilation greater than five days (OR = 6.3; 95% CI = 1.5-12.1; p = 0.024), loop diuretics (OR = 12.7; 95% CI = 4.8-25.3; p = 0.001), PDA ligation (OR = 4.6; 95% CI = 0.73-42.4; p = 0.018), and operation for ROP (OR = 3.5; 95% CI = 1.2-11.3; p = 0.034)
Karaca et al., 2014 [25]	Turkey	Cross-sectional study for children born between 2009 and 2012	Of the 2284 infants screened, 157 had BHL, and 205 had UHL	DEOAE	Chi-square test	Vaginal delivery (p = 0.027), maternal infections (p = 0.01), consanguineous marriage of parents (p = 0.02), low birth weight (<1500 g) (p = 0.038), and hyperbilirubinemia (p = 0.05) are related risk factors
Mukherjee et al., 2013 [26]	India	Cross-sectional comparative study	Infants of six months to one year of age, having any of the risk factors at birth for delayed-onset hearing loss, recognized by the Joint Committee on Infant Hearing (study group: n = 87), and matched infants without any risk factors as controls (n = 40)	BERA in the high-risk infants	Multiple logistic regression	Out of 20 risk factors, 12 factors were examined for correlation using OR with greater than 40 dB threshold. The following factors exhibit high OR: family history (OR = 41.890), ototoxic drugs (OR = 21.421), craniofacial abnormality (OR = 20.138), microcephaly (OR = 6.886), cerebral palsy and mental retardation (OR = 5.844), and developmental delay (OR = 4.334)

Characteristics of the Selected Studies

According to Omar et al., transient-evoked OAE (TEOAE) is a noninvasive, cost-effective approach of screening newborns in the NICU that enables early hearing detection and intervention (EHDI) for children with HL [11]. In children under the age of three, Zaqqout and Hamad identified that children who experienced sporadic loud noise exposure and recurrent otitis media were more likely to develop HL (p < 0.001). In these cases, there was a higher rate of maternal unemployment and a lower percentage of females having received antenatal services [13].

In children with sensorineural, conductive, and mixed types of HL, Wroblewska-Seniuk et al. found that the prevalence of the risk factors was comparable. Sensorineural and conductive HL appear to be predisposed by hyperbilirubinemia and isolated craniofacial malformations, respectively. While conductive HL is typically of a mild degree, sensorineural HL occurs predominantly bilaterally and profoundly [21].

The severity of HL was highlighted by Abu-Shaheen et al. [23] in Jordanian newborns. Additionally, an association was found between HL and the following factors: at least one Joint Committee on Infant Hearing (JCIH) risk factor, NICU admission for five or more days, LBW, postnatal hypoxia, and forceps delivery. It has also been demonstrated by Choi et al. [15], Gohari et al. [16], Hajare and Mudhol [10], and Hardani et al. [17] that a multitude of factors influence the HL of infants seeking treatment in the NICU.

According to Eras et al., preterm infants who underwent surgery for premature retinopathy also had higher levels of HL [24]. Karaca et al. assessed the prevalence of risk factors and their impact on infants' evoked OAE and concluded that UNHS would be beneficial for developing evidence-based discipline [25]. Mukherjee et al. showed that high-risk infants had a high prevalence of HL by the time they were one year old, establishing the need for an EHDI of infants who may be at potential risk in developing nations such as India [26].

According to Araujo et al., dwellings for infants with risk factors for hearing loss are deemed to be moderately adequate or less than adequate and statistically have fewer affordances than residences for infants without the risk factors. As a result, it emphasizes how crucial a good environment is for promoting a child's development of motor skills [19]. Salvago et al. recommended a prudent cochlear implantation in newborns and noted that NICU children with sensorineural hearing loss (SNHL) reported worse prognoses and delayed maturation rates [12].

Using automated ABR (AABR) at 30, 45, and 70 dB, Labaeka et al. monitored 201 newborns in the NICU. It was determined that severe BHL is a frequent condition among high-risk newborns and can persist for up to six weeks after delivery [20]. In order to determine the relationship between the JCIH risk factors and the etiology of HL, Niu et al. conducted a retrospective study involving 296 children. JCIH risk exposure and etiology vary between BHL and UHL in a way that was only discernible in 42.5% of BHL and 46.7% of UHL, respectively [18].

In a retrospective analysis, Jeong et al. contrasted the risk factors in the HL group with those in the control group. Significant neonatal and maternal risk factors included were brain lesion, the use of ototoxic drugs, NICU admission spanning more than five days, and HL in the mother at delivery [14]. In order to prevent the disability and improve the quality of life, Poonual et al. screened 3120 infants aged three months using OAE and recommended that all infants be screened for hearing before 36 weeks [22].

Risk of Bias (RoB) Within Studies

Twelve cross-sectional studies and four longitudinal studies that were subjected to quality assessment using the NOS (Table 2) and JBI (Table 3) critical appraisal tools, respectively, were found to be of good quality.

**Table 2:** Quality assessment of cross-sectional studies using the JBI critical appraisal checklist Y, yes; N, no; JBI, Joanna Briggs Institute

Author and year	Hajare and Mudhol, 2022 [10]	Omar et al., 2022 [11]	Zaqqout and Hamad, 2022 [13]	Choi et al., 2020 [15]	Gohari et al., 2020 [16]	Hardani et al., 2020 [17]	Araujo et al., 2019 [19]	Wroblewska-Seniuk et al., 2018 [21]	Abu-Shaheen et al., 2014 [23]	Eras et al., 2014 [24]	Karaca et al., 2014 [25]	Mukherjee et al., 2013 [26]
Were the criteria for inclusion in the sample clearly defined?	Y	Y	Y	Y	Y	Y	Y	Y	Y	Y	Y	Y
Were the study subjects and the setting described in detail?	Y	Y	Y	Y	Y	Y	Y	Y	Y	Y	Y	Y
Was the exposure measured in a valid and reliable way?	Y	Y	Y	Y	Y	Y	Y	Y	Y	Y	Y	Y
Were objective, standard criteria used for the measurement of the condition?	Y	Y	Y	Y	Y	Y	Y	Y	Y	Y	Y	Y
Were confounding factors identified?	Y	Y	Y	Y	Y	Y	Y	Y	Y	Y	Y	Y
Were strategies to deal with confounding factors stated?	Y	Y	Y	Y	Y	Y	Y	Y	Y	Y	Y	Y
Were the outcomes measured in a valid and reliable way?	Y	Y	N	Y	Y	Y	Y	Y	Y	Y	Y	Y
Was appropriate statistical analysis used?	N	Y	Y	N	N	N	N	N	Y	Y	N	Y
Total score	7	8	7	7	7	7	7	7	8	8	7	8

**Table 3:** Quality assessment of longitudinal studies using the Newcastle-Ottawa Scale

Author and year	Salvago et al., 2022 [12]	Jeong et al., 2021 [14]	Niu et al., 2020 [18]	Labaeka et al., 2018 [20]	Poonual et al., 2016 [22]
Selection					
Representativeness of the exposed cohort	*	*	*	*	*
Selection of the nonexposed cohort	*	*	*	*	*
Ascertainment of exposure	*	X	*	*	*
Demonstration that the outcome of interest was not present at the start of the study	*	*	*	*	*
Comparability					
Comparability of cohorts on the basis of the design or analysis controlled for confounders	**	*	*	**	*
Outcome					
Assessment of outcome	*	*	X	*	*
Was follow-up long enough for outcomes to occur?	*	*	*	*	X
Adequacy of the follow-up of cohorts	*	*	*	*	*
Total score	9	7	7	9	7
Overall grade	Good	Good	Good	Good	Good

Discussion

The findings of the review revealed a wide variety of maternal and neonatal factors that have an impact on HL in children. Studies that were included in this review were conducted in a number of different countries, which include India [10,26], Egypt [11], Korea [14,15], Iran [16,17], Gaza [13], Jordan [23], Nigeria [20], Poland [21], Sweden [18], Thailand [22], Italy [12], Brazil [19], and Turkey [24,25]. Overall, the majority of these studies have appraised the role of a variety of maternal and neonatal factors related to HL.

Genetic factors are considered the most frequent cause (50%) of permanent congenital sensorineural and mixed HL followed by congenital cytomegalovirus infection (5%-20%) and structural malformations of the temporal bones (30%-40%). Premature birth raises the risk of HL, which declines with increasing gestational age and birth weight (1.2%-7.5% for babies born at 24-31 weeks and 1.4%-4.8% for babies weighing 750-1500 g). The combinations of hyperbilirubinemia, sepsis, neonatal meningitis, necrotizing enterocolitis, prolonged ventilation, and ototoxic medication also enhance the risk of HL related to the NICU [27]. The most common type of HL, affecting 1-2 out of every 1000 newborns and an additional one out of every 1000 teenagers, is sensorineural hearing loss [28].

OAE tests and ABR tests are excellent choices for screening examinations since they can be carried out at a very young age [22]. The diagnosis depends on a standard TEOAE and an absent or markedly abnormal ABR. Henceforth, it was established that ABR must be the preferred approach for a newborn hearing screening [25]. While the ABR was used for the patient assessment in the majority of the reviewed studies [10,12,15-18,20,21,24], AABR is a rapid and widely employed technique for diagnosing and screening HL [29] and was used in four of the studies reviewed [10,16,17,26]. OAE was used in four studies [21,22,24,25], five studies [11,12,16-18] used TEOAE, and three studies utilized distortion product otoacoustic emission (DPOAE) [10,23,25]. On the other hand, Salvago et al. [12] used behavioral observation audiometry or visual reinforcement audiometry, and Hardani et al. employed auditory steady-state responses [17]. Araujo et al. used the affordances in the home environment for the motor development-infant scale (AHEMD-IS), a simplistic, useful, and self-administered questionnaire that is suitable for children between the ages of three and eighteen months [19].

Mostly, logistic regression analysis [11-14,20,22-24], chi-square tests [10,15-17,19,21,25], and the frequency distribution of descriptive statistics [10,17,18] were used to analyze the relationship between HL and risk factors. In the study done by Hajare and Mudhol, the receiver operating characteristic curve was used [10]. Four of the studies that were reviewed used the Apgar score [10,16,17,22]. A low Apgar score indicates protracted mechanical ventilation and perinatal hypoxia. It has also been shown that HL in NICU babies is significantly correlated with low Apgar scores [2]. Even though UHL accounts for 20%-50% of all congenital HL, there is scant research on the outcome measures of early-onset UHL. This restricts the approaches in which healthcare planning and policy-making can be addressed [30].

EHDI practices, which are attempted with a newborn hearing screening, lead to early intervention and have significant effects on the quality of life of the children. The average age of diagnosis has decreased substantially as a result of UNHS, which is essential for optimal speech and cognitive development [31,32]. The JCIH suggests that all newborns undergo a hearing test no later than one month of age. If a child fails a hearing test, a thorough audiological assessment should be performed within three months of birth, and any identified HL should be treated promptly within six months of age with proper interventions [33,34]. A fairly normal acquisition of vocal speech has been made possible by UNHS, supplemented by conclusive audiological diagnostic methods, early hearing aid or cochlear implant fitting, and hearing rehabilitation [28].

## Conclusions

The current systematic review revealed that the primary statistically significant risk factors for HL included ventilator support, craniofacial anomalies, LBW, forceps delivery, loop diuretics, meningitis, asphyxia, intensive care, consanguinity, sepsis, the existence of at least one JCIH risk factor, Apgar scores, and hyperbilirubinemia. It is necessary to conduct additional systematic reviews to determine whether other factors that were evaluated in each study included in this review can be used to predict or increase the risk of developing HL.

## References

[1] Sohal KS, Moshy JR, Owibingire SS, Shuaibu IY (2020). Hearing loss in children: a review of literature. J Med Sci.

[2] Raeisi R, Moradi A, Rahmani K, Ameri P, Shalchi Z (2022). Risk factors for hearing loss in infants: a systematic review. J Adv Med Biomed Res.

[3] Gifford KA, Holmes MG, Bernstein HH (2009). Hearing loss in children. Pediatr Rev.

[4] Vos B, Noll D, Pigeon M, Bagatto M, Fitzpatrick EM (2019). Risk factors for hearing loss in children: a systematic literature review and meta-analysis protocol. Syst Rev.

[5] Butcher E, Dezateux C, Cortina-Borja M, Knowles RL (2019). Prevalence of permanent childhood hearing loss detected at the universal newborn hearing screen: systematic review and meta-analysis. PLoS One.

[6] Hicks KL, Robler SK, Platt A, Morton SN, Egger JR, Emmett SD (2023). Environmental factors for hearing loss and middle ear disease in Alaska native children and adolescents: a cross-sectional analysis from a cluster randomized trial. Ear Hear.

[7] Bielecki I, Horbulewicz A, Wolan T (2011). Risk factors associated with hearing loss in infants: an analysis of 5282 referred neonates. Int J Pediatr Otorhinolaryngol.

[8] Stang A (2010). Critical evaluation of the Newcastle-Ottawa scale for the assessment of the quality of nonrandomized studies in meta-analyses. Eur J Epidemiol.

[9] Joanna Briggs Institute (2017) (2017). Checklist for analytical cross sectional studies. https://jbi.global/sites/default/files/2019-05/JBI_Critical_Appraisal-Checklist_for_Analytical_Cross_Sectional_Studies2017_0.pdf.

[10] Hajare P, Mudhol R (2022). A study of JCIH (Joint Commission on Infant Hearing) risk factors for hearing loss in babies of NICU and well baby nursery at a tertiary care center. Indian J Otolaryngol Head Neck Surg.

[11] Omar KM, Mohamed ES, Abdel E, Said F, Abdelaziz NH, Aly MA (2022). Targeted newborn hearing screening in the neonatal intensive care unit of Assiut University Hospital. Egypt J Otolaryngol.

[12] Salvago P, Immordino A, Plescia F, Mucia M, Albera A, Martines F (2022). Risk factors for sensorineural hearing loss and auditory maturation in children admitted to neonatal intensive care units: who recovered?. Children (Basel).

[13] Zaqqout RF, Hamad BA (2022). Risk factors for hearing impairment in infants and toddlers in the Gaza governorates: a case-control study. Lancet.

[14] Jeong J, Youk TM, Oh J, Eo TS, Choi HS (2021). Neonatal and maternal risk factors for hearing loss in children based on population-based data of Korea. Int J Pediatr Otorhinolaryngol.

[15] Choi KY, Lee BS, Choi HG, Park SK (2020). Analysis of the risk factors associated with hearing loss of infants admitted to a neonatal intensive care unit: a 13-year experience in a university hospital in Korea. Int J Environ Res Public Health.

[16] Gohari N, Farahani F, Gharebaghy S, Alaei S, Ahmadi S, Mozafari Z (2020). The prevalence of hearing loss in infants hospitalized in the neonatal intensive care units. Aud Vestib Res.

[17] Hardani AK, Goodarzi E, Delphi M, Badfar G (2020). Prevalence and risk factors for hearing loss in neonates admitted to the neonatal intensive care unit: a hospital study. Cureus.

[18] Niu K, Brandström A, Skenbäck S, Duan M, Uhlén I (2020). Risk factors and etiology of childhood hearing loss: a cohort review of 296 subjects. Acta Otolaryngol.

[19] Araujo DM, Santos DC, Lima MC (2019). Home environment of infants with risk indicators for hearing loss tends to be less stimulating. Int J Pediatr Otorhinolaryngol.

[20] Labaeka AA, Tongo OO, Ogunbosi BO, Fasunla JA (2018). Prevalence of hearing impairment among high-risk newborns in Ibadan, Nigeria. Front Pediatr.

[21] Wroblewska-Seniuk K, Dabrowski P, Greczka G, Szabatowska K, Glowacka A, Szyfter W, Mazela J (2018). Sensorineural and conductive hearing loss in infants diagnosed in the program of universal newborn hearing screening. Int J Pediatr Otorhinolaryngol.

[22] Poonual W, Navacharoen N, Kangsanarak J, Namwongprom S (2016). Risk factors for hearing loss in infants under universal hearing screening program in northern Thailand. J Multidiscip Healthc.

[23] Abu-Shaheen A, Al-Masri M, El-Bakri N, Batieha A, Nofal A, Abdelmoety D (2014). Prevalence and risk factors of hearing loss among infants in Jordan: initial results from universal neonatal screening. Int J Audiol.

[24] Eras Z, Konukseven O, Aksoy HT (2014). Postnatal risk factors associated with hearing loss among high-risk preterm infants: tertiary center results from Turkey. Eur Arch Otorhinolaryngol.

[25] Karaca CT, Oysu C, Toros SZ, Naiboǧlu B, Verim A (2014). Is hearing loss in infants associated with risk factors? Evaluation of the frequency of risk factors. Clin Exp Otorhinolaryngol.

[26] Mukherjee SS, Mukherjee S, Sarkar KD (2013). Prevalence of hearing loss in high risk infants of mediocre socio-economic background at around one year of age and their correlation with risk factors. Indian J Otolaryngol Head Neck Surg.

[27] Lieu JE, Kenna M, Anne S, Davidson L (2020). Hearing loss in children: a review. JAMA.

[28] Wrobel C, Zafeiriou MP, Moser T (2021). Understanding and treating paediatric hearing impairment. EBioMedicine.

[29] Warasanti ES, Purnami N, Soeprijadi S (2020). Comparison results of automated auditory brainstem response and brainstem evoked response audiometry for hearing loss detection in high-risk infants. Open Access Maced J Med Sci.

[30] Laugen NJ, Erixon E, Huttunen K, Mäki-Torkko E, Löfkvist U (2021). Newborn hearing screening and intervention in children with unilateral hearing impairment: clinical practices in three Nordic countries. J Clin Med.

[31] Joint Committee on Infant Hearing (2019). Year 2019 position statement: principles and guidelines for early hearing detection and intervention programs. J Early Hear Detect Interv.

[32] Rajendran V, Roy FG (2011). An overview of motor skill performance and balance in hearing impaired children. Ital J Pediatr.

[33] Biswas AK, Goswami SC, Baruah DK, Tripathy R (2012). The potential risk factors and the identification of hearing loss in infants. Indian J Otolaryngol Head Neck Surg.

[34] Martínez-Cruz CF, Poblano A, Fernández-Carrocera LA (2008). Risk factors associated with sensorineural hearing loss in infants at the neonatal intensive care unit: 15-year experience at the National Institute of Perinatology (Mexico City). Arch Med Res.

